# Cost-effectiveness of using a rapid diagnostic test to screen for human African trypanosomiasis in the Democratic Republic of the Congo

**DOI:** 10.1371/journal.pone.0204335

**Published:** 2018-09-21

**Authors:** Paul R. Bessell, Crispin Lumbala, Pascal Lutumba, Sylvain Baloji, Sylvain Biéler, Joseph M. Ndung'u

**Affiliations:** 1 Epi Interventions Ltd., Edinburgh, United Kingdom; 2 Programme National de Lutte contre la Trypanosomiase Humaine Africaine, Kinshasa, République Démocratique du Congo; 3 Global Health Institute, University of Antwerp, Antwerp, Belgium; 4 Faculty of Medicine, University of Kinshasa, Kinshasa, République Démocratique du Congo; 5 Institute National de Recherche Biomédicale, Kinshasa, République Démocratique du Congo; 6 Foundation for Innovative New Diagnostics (FIND), Campus Biotech, 9 Chemin des Mines, Geneva, Switzerland; Universidade Nova de Lisboa Instituto de Higiene e Medicina Tropical, PORTUGAL

## Abstract

New rapid diagnostic tests (RDTs) for screening human African trypanosomiasis (HAT) have been introduced as alternatives to the card agglutination test for trypanosomiasis (CATT). One brand of RDT, the SD BIOLINE HAT RDT has been shown to have lower specificity but higher sensitivity than CATT, so to make a rational choice between screening strategies, a cost-effectiveness analysis is a key element. In this paper we estimate the relative cost-effectiveness of CATT and the RDT when implemented in the Democratic Republic of the Congo (DRC). Data on the epidemiological parameters and costs were collected as part of a larger study. These data were used to model three different diagnostic algorithms in mobile teams and fixed health facilities, and the relative cost-effectiveness was measured as the average cost per case diagnosed. In both fixed facilities and mobile teams, screening of participants using the SD BIOLINE HAT RDT followed by parasitological confirmation had a lower cost-effectiveness ratio than in algorithms using CATT. Algorithms using the RDT were cheaper by 112.54 (33.2%) and 88.54 (32.92%) US dollars per case diagnosed in mobile teams and fixed health facilities respectively, when compared with algorithms using CATT. Sensitivity analysis demonstrated that these conclusions were robust to a number of assumptions, and that the results can be scaled to smaller or larger facilities, and a range of prevalences. The RDT was the most cost-effective screening test in all realistic scenarios and detected more cases than CATT. Thus, on this basis, the SD BIOLINE HAT RDT could be considered as the most cost-effective option for use in routine screening for HAT in the DRC.

## Introduction

Human African trypanosomiasis (HAT) or sleeping sickness is caused by two subspecies of the protozoan parasite *Trypanosoma brucei*. This tsetse fly-transmitted disease is endemic in 36 sub-Saharan African countries. In recent years, the number of new cases of the chronic form of HAT caused by *T*.*b*. *gambiense* that were reported to the World Health Organisation (WHO) has decreased from 25,841 in 2000 to 2,131 cases in 2016. However, the actual number of cases is estimated to be much higher, as many patients remain undiagnosed or unreported [1]. The WHO roadmap on neglected tropical diseases (NTDs) of 2012 that was endorsed by the London Declaration of 2012 targets the elimination of HAT as a public health problem by 2020 [2].

A core component of HAT elimination strategies is the screening of large numbers of individuals who are at risk of infection, to identify and treat cases and break the transmission cycle [3–5]. This is because the clinical signs of HAT are non-specific, and prevalence of the disease in most regions is relatively low [6]. Furthermore, due to the relative toxicity of the drugs used and the onerous nature of the treatment, it is important to correctly identify infected individuals before they are given treatment.

Screening for HAT has traditionally been carried out using the card agglutination test for trypanosomiasis (CATT) [7]. Recently, two rapid diagnostic tests (RDT) have been commercialised and are being introduced in several endemic countries [8–10]. The RDT has advantages of being simple, easy to use, not requiring electricity, is instrument-free, and has a higher sensitivity than CATT when performed on whole blood (CATT_WB_) but the RDT has lower specificity than CATT_WB_, meaning that more false positives are identified during screening [11]. Some testing algorithms include performing CATT on serial dilutions of plasma, which improves specificity, but results in some cases being missed [7,12]. Furthermore, the current format of packaging makes it bulky to transport. Individuals who are positive by CATT_WB_ or RDT (and referred to as screening suspects) must undergo further tests to confirm or rule out disease using a combination of parasitological tests. These consist of methods to identify parasites in various body fluids by microscopy, including blood, lymph node aspirates and the cerebrospinal fluid (CSF) [13]. After confirmation of disease, the CSF of HAT cases is examined by microscopy in a process known as staging, in order to determine the treatment to be used. In the DRC, routine parasitological tests comprise examination of lymph node aspirate (LN), micro-haematocrit centrifugation technique (mHCT) and the miniature anion exchange centrifugation technique (mAECT). In the DRC, active screening is carried out by mobile teams that visit communities in areas of high incidence. Although the majority (around 74%) of the local population typically presents for screening [6,14,15], this varies greatly between populations [6] and some high risk individuals may be missed [5]. Upon confirmation and staging, cases are referred to the nearest health facility that offers treatment. Individuals that are suffering from an illness may present to the local health facility, and if HAT is clinically suspected, they are screened at that facility or are referred to the nearest facility that can screen for HAT. As prevalence declines and active screening programs are reduced, passive screening is becoming an increasingly important method of case detection in the DRC [7,8].

In view of the reported advantages and disadvantages of using either RDTs or CATT, it is vital to establish the relative cost-effectiveness of implementing different algorithms in different settings [16].

## Materials and methods

A study was carried out in the DRC in 2013 to evaluate the performance of three algorithms for diagnosis of HAT during active screening by mobile teams and passive screening at fixed health centres [11]. Two algorithms used either the SD BIOLINE HAT RDT or CATT_WB_ for screening, followed by a combination of routine microscopy tests. In the third algorithm, CATT_WB_ was used for screening, and positives tested by CATT on plasma diluted 1:8. During the study, the tests were performed in parallel and the performance of algorithms subsequently evaluated. These testing algorithms were performed by four mobile teams and four hospitals and health facilities, located in three provinces in the DRC. From 16,480 people screened, 131 HAT cases were confirmed, and 13,526 controls identified [11].

We developed a model to calculate the number of cases diagnosed and the total costs of running three different HAT diagnostic algorithms in health facilities and mobile teams over one calendar year. The model was populated using epidemiological parameters and costs, and these were further tested using a number of sensitivity analyses.

In the model framework, the costs are estimated from the societal perspective, which includes all costs that are absorbed in the DRC, and excludes subsidies that are applied externally. The model we have developed evaluates the costs of screening, diagnosis and staging.The model does not include costs of treatment but for illustrative purposes, we evaluate the cost per DALY averted as if the diagnosed cases were treated, given the published treatment efficacy.

### Model structure

Three testing algorithms that are currently in use in the DRC are modelled in this analysis, as summarised below. The parasitological testing algorithm is that in routine use in DRC (LN-mHCT-mAECT):

1. Screening with CATT_WB_, confirmation by a combination of parasitological techniques and staging.
2. Screening with CATT_WB_, further screening of positives by CATT_WB_ using CATT on diluted plasma, Confirmatory testing of patients positive at a 1:8 dilution by a combination of parasitological techniques and staging.
3. Screening with RDT, confirmation by a combination of parasitological techniques and staging.

Estimation of the cost-effectiveness requires test and disease parameters as well as costs at different levels. The costs included comprise those of materials for performing the screening tests and the diagnostic tests, the capital costs for equipment, fixed costs such as staff wages, recurrent costs (annual and daily) and costs incurred by the individuals that presented for screening (travel costs and income foregone).

Thus, for each algorithm we considered:

1. The number of people that presented for testing, the cost of administering the screening test (materials and staff time) and the cost incurred by the individual to attend the test.
2. Based on disease prevalence and screening test accuracy parameters, the patients were grouped into four categories following screening:
  Truepositives=Numberscreenedxprevalencextestsensitivity
  Falsenegatives=Numberscreenedxprevalencex(1‑testsensitivity)
  Falsepositives=Numberscreenedx(1‑prevalence)x(1‑testspecificity)
  Truenegatives=Numberscreenedx(1‑prevalence)xtestspecificity
3. False positives and true positives from screening were tested for the presence of parasites using the LN-mHCT-mAECT microscopy algorithm. True positives were tested sequentially with microscopy methods until parasites were demonstrated, or if parasites were not demonstrated, then the patient was a false negative (in addition to the screening false negatives). False positives were tested with the complete microscopy algorithm in order to confirm the absence of parasites. We accounted for the costs of the microscopy tests conducted (materials and staff time).

### Assumptions

A number of rules and assumptions underpinning this model are detailed below:

1. Mobile teams are dedicated solely to diagnosis of HAT, while fixed facilities are involved in managing multiple diseases and medical conditions. Therefore, for mobile teams we consider the total cost for staffing and maintaining a mobile team for one year, whereas for fixed health facilities we consider the proportion of staffing resources that are used to test and confirm each HAT suspect and case.
2. The prevalence of HAT observed by mobile teams is an estimate of the "true prevalence", meaning that we assume it to be a representative sample of the local population at risk. This differs from the prevalence observed by fixed health facilities, where patients are screened based on a prior probability of infection. In fixed health facilities patients are selected either because they present due to symptoms or based on clinical suspicion established through consultation with a doctor or nurse. Therefore, we refer to detection at fixed health facilities as a case detection rate.
3. Diagnostic tests used for parasitological confirmation in these algorithms are independent. Data from a recent study of confirmatory tests in the DRC showed that the system sensitivity of LN-mHCT-mAECT was 77.9%, but if the tests were independent, the sensitivity would have been 90.0% [17], the sensitivity may also be boosted by CSF examination, when this is performed on the basis of clear clinical signs.
4. The combination of microscopy tests for confirming cases has 100% specificity. As the tests are all based on visualization of parasites, this is a reasonable and widely accepted assumption [14,18,19]. However, there is the potential for occasional operator error [20].
5. The RDT performed on whole blood and the CATT on serial dilutions are independent tests.
6. All the CATT reagents are used by mobile teams. The CATT is packaged such that one vial of CATT reagent is used to perform 50 tests. Once opened the reagent must be stored at 4–8°C and re-tested each day on positive and negative control samples and can get spoiled. In the case of mobile teams, we assume that at the end of each day, the remaining CATT reagent is stored and retains its potency until the following day. Therefore, we assume that 100% of available CATT tests are used by mobile teams.
7. All cost calculations are in US dollars (USD), and where necessary, converted using the 2013 exchange rates (S1 File).
8. Costs are considered from the perspective of the DRC, and only costs incurred within the DRC are included. The purchase (pre-shipment) cost of the SD BIOLINE HAT RDT to the DRC is 0.5 USD after a subsidy of 0.25 USD that is borne externally. In the study we consider the purchase cost of 0.5USD and this subsidy on RDTs is examined in sensitivity analysis.
9. Due to the packaging of the RDT, its transportation presents a challenge. Accordingly, we assumed that additional transportation equipment was required for the algorithm involving RDTs in mobile teams and include costs accordingly.

### Diagnostic test and epidemiological parameters

Wherever possible, parameters for sensitivity and specificity of diagnostic tests were taken from a clinical trial of the SD BIOLINE HAT RDT [11] and compared to other published estimates (Table 1). We placed an emphasis on alternative estimates that are from the DRC whenever these were available, particularly on data from the national HAT control program in the DRC (PNLTHA). Any parameter estimated from the SD BIOLINE HAT RDT clinical trial that differed markedly from previously published estimates, or was subject to variability, was tested in sensitivity analysis.

**Table 1:** Diagnostic tests and epidemiological parameters.

Parameter	Estimate (95% CIs)*	Alternative observations (source)	Comments (sources)
HAT prevalence	0.82% (0.68–0.98)	0–2% (PNLTHA)	Based on case detection rates at active screening
HAT incidence rate among individuals reporting for passive screening	1.76% (1.28–2.42)	0–5% (PNLTHA)	
Proportion of HAT cases in stage 2 Health facilities Mobile teams	50.0% (33.6–66.4) 22.2% (15.2–31.4)		
Number presenting for screening to the mobile teams	250 per day	120–350 (PNLTHA)	
Number presenting for screening at health facilities	10 per day	1–30 (PNLTHA)	Smaller facilities will test fewer individuals
Sensitivity RDT	92.0% (86.1–95.5)		
Specificity RDT	97.1% (96.8–97.3)		
Sensitivity CATT_WB_	69.1% (60.7, 76.4)	87% - 98% [6]	91.2% used for comparison [19]
Specificity CATT_WB_	98.0% (97.8–98.3)	83.5–99.3% [7]	97.4% used for comparison [19]
Sensitivity CATT on 1:8 diluted plasma	59.2% (50.4–67.4)		77.6% used for comparison [19]
Specificity CATT on 1:8 diluted plasma	99.6% (99.5–99.7)		99.1% used for comparison [19]
Proportion with palpable lymph nodes HAT cases HAT suspects	39.7% (31.7–48.3) 11.7% (9.3–14.6)		.
Sensitivity LN	71.2% (57.7–81.7)		Sensitivity among those with palpable lymph nodes. The overall sensitivity is 28.2%.
Sensitivity mHCT	55.2% (45.3–64.8)	56.5% [14] 52.1% [17]	
Sensitivity mAECT	78.3% (66.4–86.9)	75.3% [14] 68.1% [17]	
Efficacy stage 1 treatment (Pentamidine)^#^	99.0%		Source [14]
Efficacy stage 2treatment (NECT)^#^	96.0%		Source [21]
Iatrogenic mortality stage 1 treatment (Pentamidine)^#^	0.1%		Source [14]
Iatrogenic mortality stage 2 treatment (NECT)^#^	1.0%		Source [21]

*Data were sourced from the SD BIOLINE HAT RDT clinical trial [11], unless otherwise stated.

^#^ These are included for the estimation of DALYs averted.

In this model, mobile teams screen for 220 days per year, by working for 20 days followed by 10 days of rest each month for 11 months in a year, with one full month off duty. Fixed facilities operate for 250 days per year, consisting of 52 working weeks and 10 public holidays. To calculate the payment per hour from an annual salary, we assume that staff work 2,000 hours per year. This is based on an average of 40 hours per week for 52 weeks minus 10 public holidays.

### Costs

Details of the derivation and calculation of the costs used in these analyses are given as supplementary information (S1 File). The costs are broken down as follows:

1. Capital costs: These are the costs of purchasing the equipment necessary to run the mobile team or the HAT screening at a fixed unit. Capital costs are calculated using the straight line depreciation method over a 5-year useful life for mobile teams and 20 years for fixed health facilities. Total annual capital costs for a mobile team were 12,001 and 12,781 USD for mobile teams implementing CATT and RDTs respectively, and 551 and 496 USD for a fixed health facility for CATT and RDTs respectively.
2. Annual recurrent costs: For mobile teams, these include the costs of staffing the unit for one year (staffing costs for fixed health facilities are calculated on a per test basis), the costs of training, insurance and maintenance. Total costs for mobile teams are 30,307 USD / year and 435 USD / year for fixed health facilities.
3. Daily running costs: These include the costs of consumables such as fuel, water and stationery. For mobile teams, they also include the allowances paid to staff. For mobile teams, the total costs are 97 USD per screening day (21,340 USD / year) and for fixed health facilities 3.50 USD per screening day (875 USD / year or 0.35 USD per patient screened, based on screening 10 patients per day).
4. Staffing costs for fixed health facilities: The cost for a laboratory technician is 0.78 USD / hour and 0.63 USD / hour for a nurse based on national salaries. We assume that the consultation at a fixed health facility is with a nurse, although in practice this could occasionally be with a doctor.
5. The costs of materials (including shipping) for the diagnostic tests are: CATT_WB_ is 0.70 USD in mobile teams and 0.76 USD in fixed facilities (the cost at the fixed facility includes a small loss due to repeating control tests), RDT 0.60 USD (0.85 excluding a 0.25 USD subsidy, but including 0.1 USD shipping cost), CATT dilutions 3.02 USD; LN 0.38 USD; mHCT 1.54 USD; mAECT 7.20 USD; materials for CSF examination are 2.00 USD.
6. The time required to perform each test was (in minutes): clinical consultation 20; CATT_WB_ 10; RDT 17; CATT dilutions 15; aspiration of lymph nodes and examination of aspirate 15; mHCT 18; mAECT 30; examination of CSF 30.
7. We included costs incurred by individuals presenting for screening, including travel costs and costs of missed work. These were 0.32 USD for mobile teams and 1.97 USD for fixed health facilities.

### Implementation and analysis

The model is implemented in the R statistical environment [22]. From the model, the following values are calculated:

1. The total cost of implementing the surveillance activities for one year.
2. The number of people screened in one year.
3. The number of people that were infected with HAT among the number screened.
4. The number of false positives from screening that then undergo confirmatory testing by microscopy.
5. The number of false negatives after screening and confirmatory testing (missed cases), this comprises both false negatives from screening that did not proceed for confirmatory testing as well as false negatives following confirmatory testing.
6. The number of HAT cases that were diagnosed.
7. For the estimation of DALYs the number of HAT cases that are subsequently cured assuming that all cases present for treatment, and considering the published treatment efficacy. This is calculated as the number of true positives that were successfully treated (number of confirmed HAT cases in each stage of HAT that were successfully treated given the treatment efficacy, and the iatrogenic mortality due to treatment for stage 1 and stage 2 of HAT). The corresponding number of disability adjusted life years (DALYs) averted is calculated, considering 28.6 and 25.1 DALYs averted for a true positive treated in stage 1 and stage 2 respectively [23] (S1 File).

From this we calculate the average cost-effectiveness ratio (ACER) as the total cost per HAT case diagnosed, and the incremental cost-effectiveness ratio (ICER) as the difference between each algorithm and the algorithm that was most cost-effective by ACER. We also calculate the cost per disability adjusted life year (DALY) averted, considering 28.6 and 25.1 DALYs averted for a true positive treated in stage 1 and stage 2 respectively [23]. The DALYS are discounted and age weighted, calculated using life expectancies from the DRC (S1 File).

### Sensitivity analysis

A number of sensitivity analyses are carried out to test the robustness of the model to the parameters outlined above. Specifically, we test:

1. A range of sensitivities of the CATT_WB_ and RDT from 60–100% and specificities from 90–100%.
2. Revised CATT_WB_ sensitivity of 91.2% and specificity of 97.4% based on a review [19]. Accordingly, the sensitivity of CATT at 1:8 dilution following a positive CATT_WB_ test is 85.1% and specificity 63.6% following a positive CATT_WB_ test [19].
3. Revised RDT and CATT sensitivity and specificity based on a recent paper comparing three screening tests [24]. The paper found comparable specificities but considerably lower sensitivities estimated separately for passive and active screening. The sensitivities of the RDT were 49.2 and 70% in active and passive screening respectively. For CATT they were 51.8 and 74.6% respectively. Specificities of the RDT were 99.4 and 96.7% in active and passive screening and for CATT 99.5% and 97.6% respectively. The study also included a second generation of RDT with a sensitivity in active screening of 54.8% and 90.1% in passive screening. Corresponding specificities are 99.1% and 93.7%. This second generation RDT has the same purchase cost of 0.5USD.
4. The cost of the RDT without the subsidy of 0.25 USD.
5. Prevalence of HAT among the population that presents for screening ranging from 0.1% - 2%.
6. The number presenting for screening at fixed health facilities, from 1 patient per day (to represent smaller facilities) to 30 patients per day to represent the largest hospitals in highly endemic areas.
7. Variations in the numbers presenting daily for screening to a mobile team from 120 to 350.
8. An additional cost to the patient for presenting for confirmation from fixed health facilities, to reflect algorithms that are used in Uganda and Kongo Central in DRC [8,10] where patients may be referred for confirmation. Supplementary sums ranging from 1–20 USD are considered.

Of the 7 analyses listed above, only the first three will influence the dominance of one algorithm over another. The remainder change the overall costs of all algorithms equally.

### CHEERS checklist

The analyses is consistent with the Consolidated Health Economic Evaluation Reporting Standards (CHEERS) guidelines [25,26]. The completed checklist is S1 Table.

### Ethics statement

The protocol for the clinical trial on the RDT was approved by the Ethical Review Committee of Ngaliema Clinic, Ministry of Public Health of the Democratic Republic of the Congo (approval number 184/2013). Written informed consent was obtained from each participant before enrolment in the study. In the case of children, informed consent was obtained from a parent or guardian.

## Results

The optimal algorithm measured by the ACER, by the cost per DALY averted, and by the number of deaths averted for both mobile teams and fixed health facilities is the RDT followed by a combination of microscopy tests (Table 2). In terms of the ICER, in mobile teams the algorithm using the RDT strongly dominated (it was cheaper and diagnosed more cases) the algorithm with CATT_WB_. The algorithm using the RDT weakly dominated (it cost more but diagnosed more cases) the algorithm with CATT dilutions. In fixed health facilities, the algorithm using the RDT weakly dominated both alternatives. Implementation of any of the algorithms in a fixed facility always had a better ACER than they did when implemented in a mobile team.

**Table 2:** Summary of cost-effectiveness analysis results.

Algorithm	Screening false positives (%)	Cases diagnosed (%)	HAT cases cured (%)	Total cost		USD / DALY averted		ACER	ICER
**Mobile teams; 55,000 screened; 449.5 HAT +ve**										
CATT_WB_	1080.8 (2.0)	288.9 (64.3)	283.3 (63.0)	130351			16.8	451.2	-0.876
CATT_WB_ + CATT_1:8_	220.6 (0.4)	251.5 (56.0)	246.6 (54.9)	126811			18.7	504.2	25.95
**RDT**	**1566.7 (2.8)**	**384.7 (85.6)**	**377.2 (83.9)**	**130267**			**12.6**	**338.6**	**-**
**Fixed health facilities; 2,500 screened; 44.0 HAT +ve**										
CATT_WB_	48.7 (1.9)	28.3 (64.3)	27.4 (62.3)	10115	13.4			357.6	1.98
CATT_WB_ + CATT_1:8_	9.9 (0.4)	24.6 (56.0)	23.9 (54.3)	9986	15.2			405.5	11.31
**RDT**	**70.5 (2.8)**	**37.7 (85.6)**	**36.5 (83.0)**	**10133**	**10.1**			**269.1**	**-**

The cost-effectiveness of 3 algorithms modelled in the 2 screening strategies over one year of screening. The two optimal algorithms are shown in bold. CATT_1:8_: CATT is performed on serially diluted plasma samples, using the 1:8 dilution as cut-off.

In fixed health facilities the majority of the costs are those for screening and costs incurred by patients (Table 3). In mobile teams the majority of the costs are annual costs (including staff wages) and screening (Table 3). Participant costs accounted for 48.60% and 13.51% of costs at fixed health facilities and mobile teams respectively.

**Table 3:** Cost breakdown.

	RDT + microscopy fixed health facilities			RDT + microscopy mobile team		
Description	Cost	% total cost	Cost per case diagnosed	Cost	% total cost	Cost per case diagnosed
Capital costs	496.10	4.90	13.58	12,781.20	9.81	33.89
Annual costs	435.00	4.29	11.91	30,307.00	23.27	80.36
Daily costs	875.00	8.63	23.96	21,340.00	16.38	56.58
Screening costs	2,577.50	25.44	70.58	33,000.00	25.33	87.50
Participant costs	4,925.00	48.60	134.86	17,600.00	13.51	46.67
Confirmation costs	824.75	8.14	22.58	15,238.52	11.70	40.40
	**10,133**		**277.48**	**13,0267**		**345.39**

Breakdown of the costs incurred to implement the most cost effective algorithms by mobile teams and at fixed health facilities.

### Sensitivity analysis

When the sensitivity and specificity of the CATT_WB_ is adjusted to be similar to that reported in previous studies [19] the ACER of all algorithms involving CATT closes in on that of the RDT, but the algorithm comprising the RDT remains most cost-effective in both mobile teams and fixed health facilities (Table 4).

**Table 4:** Summary of cost-effectiveness analysis results with revised parameters for CATT_WB_.

Algorithm	Screening false positives (%)	Cases diagnosed (%)	HAT cases cured (%)	Total cost		USD / DALY averted		ACER	ICER
**Mobile teams; 55,000 screened; 449.5 HAT +ve**										
CATT_WB_	1418.3 (2.6)	381.4 (84.8)	373.9 (83.2)	133671			13.0	350.5	-1037
CATT_WB_ + CATT_1:8_	516.3 (0.9)	332.0 (73.9)	325.5 (72.4)	131024			14.7	394.6	-14.38
**RDT**	**1566.7 (2.8)**	**384.7 (85.6)**	**377.2 (83.9)**	**130267**			**12.6**	**338.6**	**-**
**Fixed health facilities; 2,500 screened; 44.0 HAT +ve**										
CATT_WB_	63.9 (2.6)	37.3 (84.8)	36.2 (82.3)	10297	10.4			275.8	-507.7
CATT_WB_ + CATT_1:8_	23.2 (0.9)	32.5 (73.9)	31.5 (71.6)	10224	11.8			314.5	-17.63
**RDT**	**70.5 (2.8)**	**37.7 (85.6)**	**36.5 (83.0)**	**10133**	**10.1**			**269.1**	**-**

The cost-effectiveness of 3 algorithms modelled in the 2 screening strategies over one year of screening with a CATT_WB_ sensitivity of 91.2% and specificity 97.4%. The two optimal algorithms are shown in bold. CATT_1:8_: CATT is performed on serially diluted plasma samples, using the 1:8 dilution as cut-off.

**Fig 1:**
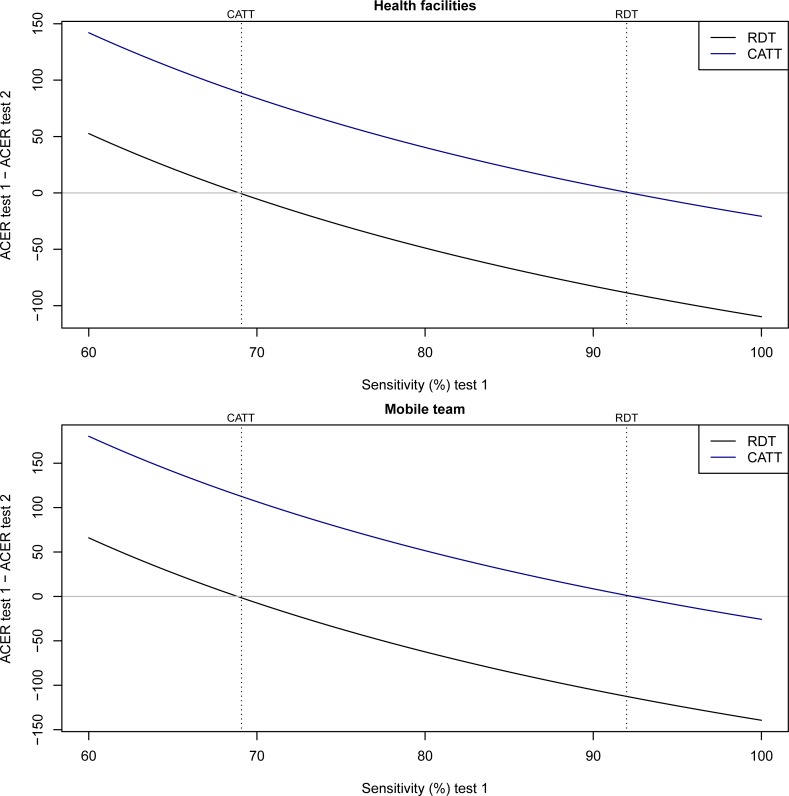
ACER at varying sensitivities. We vary the sensitivity parameter of one of the tests (test 1 = the solid lines) and show the difference in ACER between test 1 and the reference test (test 2) whose sensitivity parameter is fixed at the values in Table 1. The RDT is compared to CATT_WB_ and vice-versa. The broken lines show the sensitivity of the reference tests.

When varying the sensitivities of the two basic tests is considered at health facilities the CATT_WB_ becomes more cost-effective than the RDT if the sensitivity of the RDT is below 68.9%, or if the sensitivity of CATT_WB_ is above 92.2% (Fig 1). The corresponding values at mobile teams are 68.8% and 92.3%

**Fig 2:**
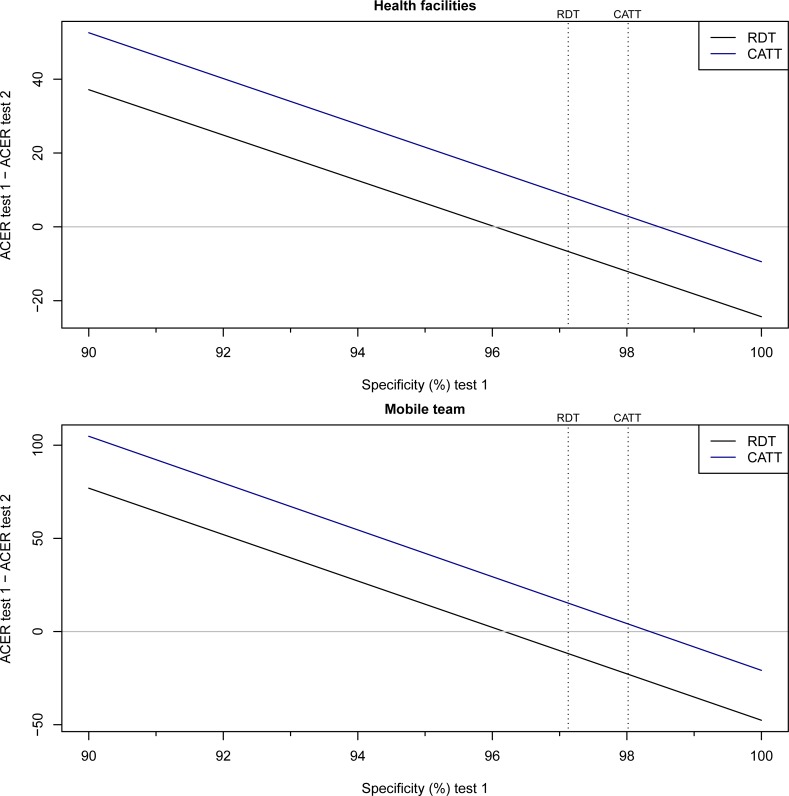
ACER at varying specificities. We vary the specificity parameter of one of the tests (test 1 = the solid lines) and show the difference in ACER between test 1 and the reference test (test 2) whose specificity parameter is fixed at the values in Table 1. The RDT is compared to CATT_WB_ and vice-versa. The broken lines show the specificity of the reference tests.

Considering screening test specificity, the CATT becomes more cost-effective at health facilities when the specificity of the RDT is below 96.0%, or the specificity of CATT is above 98.5% (Fig 2). The corresponding specificities at mobile teams are 96.2% and 98.3% (Fig 2).

Using the CATT and RDT results of [24] with a sensitivity of the RDT that is below that of CATT then the ACER of CATT in active screening is $568 and of RDT is $577. In passive screening the respective ACERs are $335 and $356. An algorithm including screening with the second generation RDT followed by confirmation and staging would have an ACER of $525 in active screening and $296 in passive screening.

When the 0.25 USD subsidy on the SD BIOLINE HAT RDT is not included, the ACER for the RDT followed by microscopy in mobile teams is 381.85 USD. It is 285.70 at fixed health facilities.

**Fig 3:**
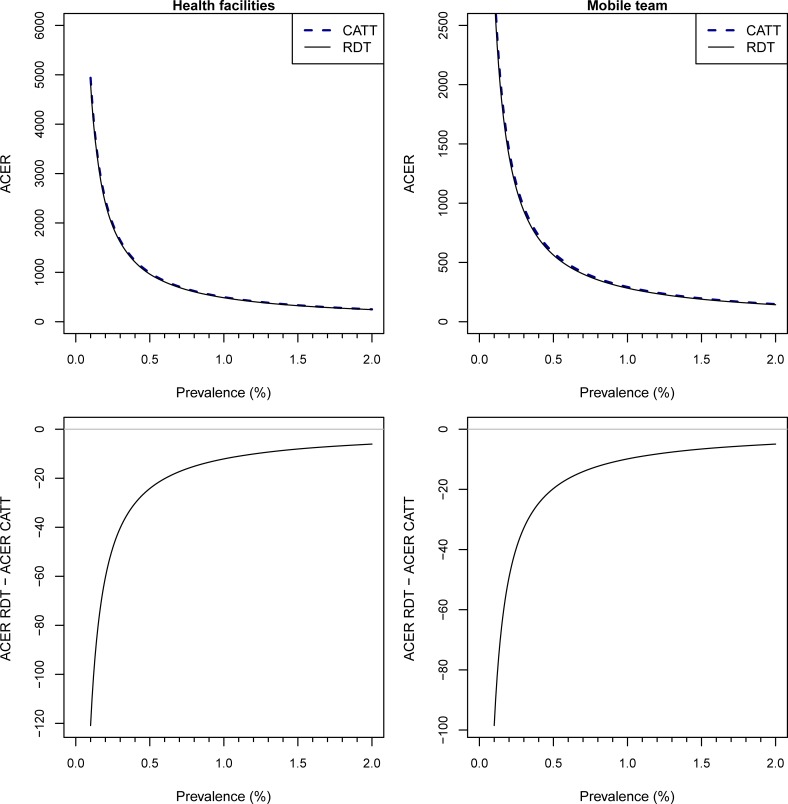
ACERs at varying HAT prevalences. We vary the prevalence parameter from 0.1–2% with the sensitivity and specificity of CATT_WB_ set at 91.2% and 97.4%, The top two plots show the ACERs of CATT_WB_ and the RDT in health facilities and mobile teams. The bottom two plots show the difference between the ACERs for the two tests at health facilities and at mobile teams.

At all plausible HAT prevalences, the RDT remains more cost-effective than CATT, but converges at higher prevalences, when the CATT parameters from [19] are used (Fig 3).

**Fig 4:**
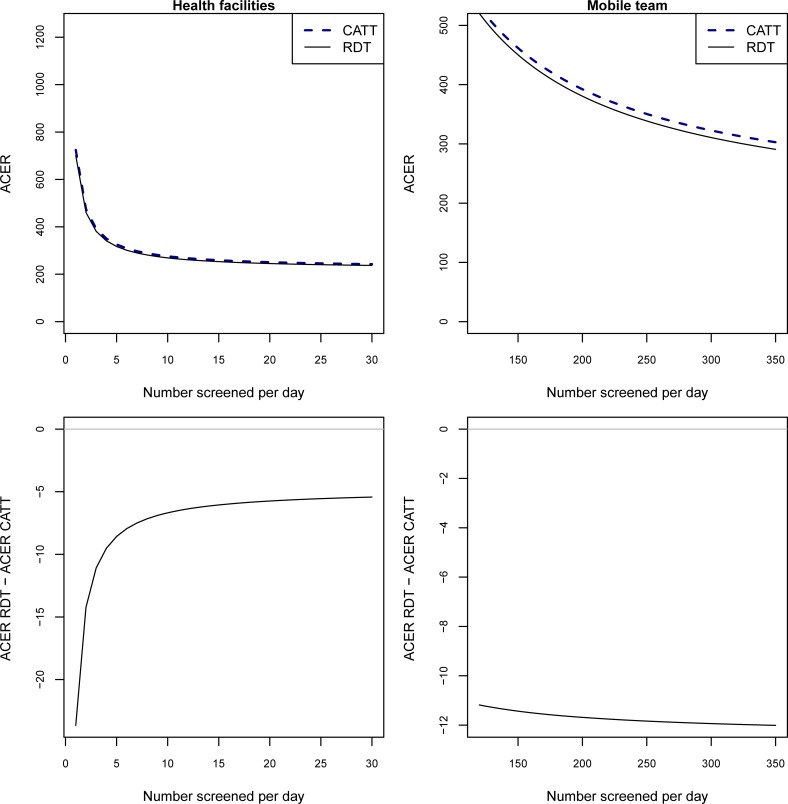
ACERs at varying numbers screened. We vary the numbers screened at health facilities from 1–30 and at mobile teams from 120–350 with the sensitivity and specificity of CATT_WB_ set at 91.2% and 97.4%, The top two plots show the ACERs of CATT_WB_ and the RDT in health facilities and mobile teams. The bottom two plots show the difference between the ACERs for the two tests at health facilities and at mobile teams.

The RDT remains optimal across a range of plausible numbers of people screened per day at both health facilities and mobile teams (Fig 4).

**Fig 5:**
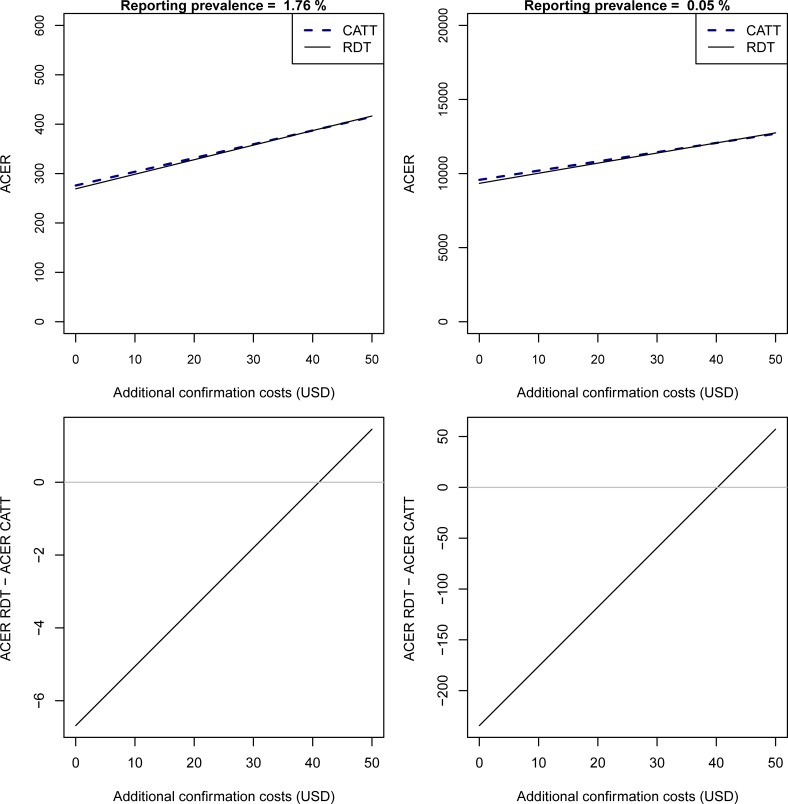
ACER at different patient confirmation costs. At different reporting prevalences at health facilities, the variation in ACER for a range of costs to a patient that is referred for confirmation with the sensitivity and specificity of CATT_WB_ set to 91.2% and 97.4%. The top plots show the ACERs for the RDT and CATT_WB_ for two different reporting prevalences and the bottom plots the difference between the ACERs.

When additional costs incurred by the patient of being referred from health facilities for confirmatory diagnosis are factored in, the RDT remains more cost-effective. This remains until the cost to the patient exceeds 41.05USD at the basic prevalence and 40.19USD at a prevalence of 0.05% (Fig 5).

## Discussion

This study demonstrates that the most cost-effective algorithm for either active or passive screening for HAT is one that includes the SD BIOLINE HAT RDT followed by microscopy. In addition to being more cost-effective, this algorithm is also the most effective in terms of the percentage of cases that are diagnosed and the DALYs that are averted. As well as being more cost-effective than CATT, the RDT has the added advantages of greater practicality by being a single use format and not requiring electricity and a cold chain [27,28]. These findings are relevant to the DRC in terms of the costs and test performance parameters.

The findings presented here are generally robust to most sensitivity analyses. The sensitivity of CATT during the clinical trial on the SD BIOLINE HAT RDT was lower than many previous estimates [19,29] but the RDT is still the most cost-effective when the analysis are re-run with the alternative CATT_WB_ parameter estimates. However, using the sensitivity and specificity estimates from a study of three tests [24] that had a sufficiently large sample size to give separate estimates for active and passive screening CATT had a marginally lower ACER than the SD BIOLINE HAT RDT in both active and passive screening. Based on this study, a second generation RDT was estimated to be more cost effective than both the first generation RDT and CATT in both active and passive screening.

When the subsidy of 0.25 USD for the SD BIOLINE HAT RDT is not considered, then the RDT is less cost-effective than CATT_WB_ at the higher sensitivity, but the RDT remains more effective in terms of the percentage of cases diagnosed. In the future, it may be possible to further reduce the costs of manufacture and shipping by modifying test format and by packing many tests in a small volume, which would be particularly attractive for active screening. Whilst difficult to quantify, the additional transportation burden of the test in its current format was allowed here by including an additional 5,000USD to the purchase cost of a vehicle.

The lower specificity of the RDT relative to CATT_WB_ increases the cost, due to higher costs of performing microscopy on the false positives identified after screening. This is principally because all screening suspects that are false positives must be tested with all microscopy methods including mAECT to confirm an absence of parasites. By using this more expensive test, the confirmation costs for the RDT algorithm were around 40% higher than those for CATT_WB_, but the RDT remained cost-effective. Despite reducing the number of individuals that proceed to the microscopy tests, CATT dilutions did not result in an improvement in the cost-effectiveness of any algorithm, due to the poor sensitivity of CATT on 1:8 diluted plasma.

As HAT nears elimination, a number of foci are adopting a passive surveillance strategy in which HAT suspects are screened at a number of facilities that perform the HAT RDT. RDT positive patients (suspects) are referred to the nearest facility with the capacity to perform confirmatory diagnosis by microscopy [8,10]. In these algorithms, the patient usually bears the cost of this referral, and therefore a less specific test such as the RDT may be less cost-effective so more false positives from screening would be referred. In these analyses, we demonstrate that this is not the case unless this cost exceeds 40USD (Fig 5).

This study has shown that active case finding by mobile teams is less cost-effective but active screening will continue to play a significant role in disease elimination. This is particularly the case in areas where there are a low number of health facilities and so the access of the population to screening is poor [30]. Conventional vehicle based active screening is also being augmented by new strategies such as targeted screening and motorcycle based screenings that may improve cost effectiveness by reducing costs or the numbers of non-infected individuals that are screened [31]. Furthermore, as active surveillance does not rely on cases becoming ill and presenting at a health facility it can identify cases earlier during infection [7,15,32]. Consequently, active case finding identifies a larger proportion of cases in stage 1 when morbidity is lower and treatment easier and safer, many infections are sub-clinical, and the patient has been infectious for a shorter period. Nevertheless, as prevalence falls, numbers presenting for active screening are also likely to fall, requiring more innovative methods of identifying cases [3,31].

## Conclusions

This study demonstrates that algorithms for screening for HAT that use the SD BIOLINE HAT RDT are the most cost-effective in both mobile teams and fixed health facilities, at the prices applicable in the DRC and sensitivities and specificities experienced there. The RDT is also the most effective in terms of case detection. Furthermore, we have provided evidence that such algorithms will remain the most cost-effective as HAT nears elimination, and the numbers presenting for screening declines.

## Supporting information

S1 File Cost and parameter derivation. This file gives details of the calculation of costs and parameters used in this study.
(DOC)

S1 Table CHEERS checklist. (DOC)
