# Supplementary material for: Cost-effectiveness of using a rapid diagnostic test to screen for human African trypanosomiasis in the Democratic Republic of the Congo
Source: PLoS One. 2018 Sep 21;13(9):e0204335. doi: 10.1371/journal.pone.0204335 (PMC6150526; doi:10.1371/journal.pone.0204335)
Supplement: S1 File — This file gives details of the calculation of costs and parameters used in this study. (DOC) [file pone.0204335.s001.doc]

**S1 File. Cost and parameter derivation**

**Supplementary information for: Cost-effectiveness of using a rapid diagnostic test to screen for human African trypanosomiasis in the Democratic Republic of the Congo**

Bessell, PR., Lumbala, C., Lutumba, P., Baloji, S., Bieler, S. and Ndung'u, JM.

This document contains additional information to describe how the costs and epidemiological parameters were derived.

**Exchange rates**

Where it was necessary to convert from non-USD currencies we used the following exchange rates from the time of the study (data from www.xe.com):

1 Euro = 1.364USD

1 Congolese Franc = 0.001106USD

**Capital costs**

Capital costs comprise the cost of tangible goods that are required to equip the fixed unit or mobile team. These costs are one-off purchases that could continue to have utility in subsequent years or be sold on, so their costs are calculated as the estimated loss of value due to depreciation in one calendar year. We calculate depreciation of the capital costs using the straight line method with no salvage value and a 5 year useful life for a mobile team’s equipment and 20 year useful life for a fixed health post – the latter to reflect the lower wear and tear at health facilities and that equipment is not necessarily dedicated to HAT screening. So the annual cost is simply the purchase cost divided by the useful life in years.

.

**Table S1**. Breakdown of the capital costs in these analyses.

| **Description** | **Capital cost** | **Annual cost (loss in value)** | **Source and comments** |
| --- | --- | --- | --- |
| Generator  Mobile team  Fixed unit | 2500.00 | 500  125 | RDT demonstration study |
| Encampment equipment and bicycle  Mobile team | 3984.00 | 797.20 | RDT demonstration study |
| Vehicle  Mobile team | 45000.00 | 9000 | Shipped cost.  PNLTHA |
| Additional transport for RDTs  Mobile team | 5000 | 1000 | Estimated |
| Furniture  Mobile team  Fixed unit | 840.00 | 168  42 | RDT demonstration study |
| Fridge and ice box  Mobile team  Fixed unit | 1290.00 | 258  64.50 | RDT demonstration study |
| Microscope  Mobile team  Fixed unit | 1992.00 | 398.40  99.60 | RDT demonstration study |
| Centrifuges  Mobile team  Fixed unit | 2300.00 | 460  115 | RDT demonstration study |
| Other lab equipment (non-consumable)  Mobile team  Fixed unit | 1000.00 | 200  50 | Estimated |
| CATT rotator (only strategies with CATT)  Mobile team  Fixed unit | 1100.00 | 220  55 | RDT demonstration study |
| **Totals**  **Mobile team (CATT)**  **Mobile team (RDT)**  **Fixed unit (CATT)**  **Fixed unit (RDT)** |  | **12001.20**  **12781.20**  **551.10**  **496.10** |  |

**Annual recurrent costs**

Annual recurrent costs comprise the costs of intangible items that are incurred on an annual basis. We consider the annual wages for mobile teams here because mobile team staff are dedicated to HAT diagnosis; for fixed units the staff costs are calculated per participant screened (Tables S4 and S6). The annual costs are listed in Table S2. Costs of training staff in the use of tests and equipment is greater at mobile teams as there are seven members of staff per team compared to the one laboratory technician to be trained at fixed units (Table S2).

**Table S2**. Annual recurrent costs incurred in these analyses.

| **Description** | **Annual cost (USD)** | **Source and comments** |
| --- | --- | --- |
| Training  Mobile team  Fixed unit | 1375.00  235.00 | PNLTHA |
| Insurance and maintenance  Mobile team  Fixed unit | 2748.00  200.00 | PNLTHA |
| Salaries  Mobile team | 26184.00 | PNLTHA |
| **Totals**  **Mobile team**  **Fixed unit** | **30307.00**  **435.00** |  |

**Daily recurrent costs**

These are the costs of maintaining and running mobile teams and health facilities for one day. For mobile teams these costs are based upon consumption of fuel, stationary and other consumables and an allowance that is paid to each staff member for each day of screening. These costs are estimated from records for a team in Kasai Oriental. For fixed units the daily maintenance costs are more difficult to ascertain as they are distributed across the operations of the unit. To estimate the cost of running just HAT diagnosis, we took the daily running costs for HGR Bagata that is USD 42. This facility has three doctors and HAT diagnosis requires 25% of the time for one doctor (based upon establishing suspicion and screening 10 cases per day). Therefore, we assumed that HAT diagnosis used one-sixth of the hospital capacity, however as this proportion is difficult to estimate with certainty and will vary greatly between facilities we explored this in the sensitivity analysis. The daily recurrent costs are broken down in Table S3.

**Table S3**. Breakdown of the daily recurrent costs in these analyses.

| **Description** | **Daily cost (USD)** | **Source and comments** |
| --- | --- | --- |
| Daily running costs  Mobile team  Fixed unit | 32.50  7.00 | PNLTHA  Hospital data |
| Allowances  Mobile team | 64.50 | PNLTHA |
| **Totals (per day of screening)**  **Mobile team**  **Fixed unit** | **97.00**  **7.00** |  |

**Diagnostic test costs**

The costs of the two staff members required for screening at fixed units are listed in Table S4. The nurse is required for a consultation to establish that the patient is an HAT suspect and the laboratory technician to perform the diagnostic tests.

**Table S4**. The costs for a doctor and laboratory technician at fixed units. The hourly rates are calculated by dividing the annual salary by 2000 (the number of hours worked in one year).

| **Description** | **Annual salary (USD)** | **Cost per hour (USD)** | **Source and comments** |
| --- | --- | --- | --- |
| Staff (Fixed units only)  Lab. technician  Nurse | 1560.00  1260.00 | 0.78  0.63 | PNLTHA |

For fixed units the cost of carrying out a diagnostic test is the cost of the materials for the test plus the cost of staff for performing the test. For mobile teams the cost is just the cost of the materials as the staff are dedicated and their costs are included in the annual recurrent costs. The cost of the materials for the test are listed in Table S5. In addition to the costs we list an estimate for the time required to conduct one test. For calculating the test cost at fixed units the time required is multiplied by the hourly rate for the staff member (laboratory technician for everything except the consultation). The consultation only applies to screening at the fixed centres. The number of times that each cost is performed and thus the total cost is derived from the epidemiological parameters.

An additional cost for the CATT and RDT is the cost of shipment and storage which for CATT includes a cost for the cold storage chain. The total cost of shipping 200,000 CATT units from production to the field is 6392 USD that is 0.04 USD per test (calculated from January 2014). Shipment and storage costs for the RDT were not available for large scale shipment and so were estimated at 0.10 USD to allow for the bulkiness of the shipment. At fixed health centres there is an additional 6 USc for the tests that are lost to repeating the control tests at the start of each screening day.

**Table S5**. The costs for conducting each of the diagnostic tests considered in this study.

| **Test** | **Time required (mins)** | **Cost (USD)** | **Source and comments** |
| --- | --- | --- | --- |
| Consultation (Doctor)  Staff costs  Total - fixed units | 20 | 1.58  **1.58** | Based upon the cost of 20 mins of a doctor's time. |
| CATT  Staff costs  Materials (Fixed unit)  Materials (mobile team)  Shipment  **Total - fixed units**  **Total - mobile teams** | 10 | 0.13  0.72  0.66  0.04  **0.89**  **0.70** | Costs at mobile teams and fixed units are different because once a bottle of CATT antigen is open repeat cases and controls must be performed. Materials are the cost of test materials plus the cost of the lancet |
| RDT  Staff costs  Materials  Shipment  **Total - fixed units**  **Total - mobile teams** | 17 | 0.22  0.50  0.10  **0.82**  **0.60** | Includes a subsidy of USD 0.25 |
| CATT titration  Staff costs  Materials  Shipment  **Total - fixed units**  **Total - mobile teams** | 15 | 0.44  2.90  **0.12**  **3.43**  **3.02** | Interviews  Cost is based on an average of three CATT dilutions ($2.10) + $0.80 for materials |
| Lymph node aspirate examination  Staff costs  Materials  **Total - fixed units**  **Total - mobile teams** | 15 | 0.19  0.38  **0.57**  **0.38** | [1] |
| CTC  Staff costs  Materials  **Total - fixed units**  **Total - mobile teams** | 18 | 0.23  1.54  **1.77**  **1.54** | [1] |
| mAECT  Staff costs  Materials  **Total - fixed units**  **Total - mobile teams** | 30 | 0.38  7.20  **7.58**  **7.20** | PNLTHA |
| Lumbar puncture examination  Staff costs  Materials  **Total - fixed units**  **Total - mobile teams** | 30 | 0.38  2.00  **2.38**  **2.00** | Estimated externally |

In addition to the costs incurred by the surveillance provider we consider the costs incurred by the individuals being screened and treated. These are shown in Table S6 and are broken down to travel and lost earnings and are different for fixed units and mobile teams as individuals typically have to travel further and take more time out of work to attend screening at a fixed unit. We have assumed that stage 1 treatment will be out-patient requiring travel for treatment for each of the 7 days of treatment but no costs of hospitalisation. Treatment for patients in stage 2 of HAT is assumed to be in-patient with travel to and from hospital, a cost of lost earnings and an additional cost of 15 USD to cover subsistence which may have to be provided by somebody else. The cost of hospitalisation is borne in full by the patient and this is included here, consistent with previous studies [3]. The period of treatment lasts for 10 days. Drugs are issued free of charge provided by donors and as stage 1 is an outpatient procedure requiring presenting to receive daily doses of oral chemotherapy we assume no cost for stage 1 treatment.

**Table S6**. The costs to the individual for screening and treatment.

| **Description** | **Mean cost per patient** | **Source and comments** |
| --- | --- | --- |
| Screening - travel  Mobile team  Fixed unit | 0.15  1.61 | Interviews with patients |
| Screening - missed work  Mobile team  Fixed unit | 0.17  0.36 | Interviews with patients |

**Calculating DALYs**

Using the methods outlined in [2] for calculating discounted and age-weighted life expectancies and the estimates of life expectancy by age band outlined in [3] we calculate the mean DALYs per HAT case. We assume that the median survival time for patients in stage 1 of HAT is 3 years and during stage 2 median survival is 1 year as per [3] and [4]. The disability adjustment factor for stage 1 is 0.191 and stage 2 was 0.81 as per [5]. The case ages used are those from the RDT demonstration study in which the ages of HAT stage 1 cases (mean = 24.72 years, n = 85) were lower than for stage 2 (mean age = 29.37 years, n = 38), although this difference is not significant.

The number of DALYs averted are calculated as:

DALY gained from treatment x proportion successfully treated - years of life lived with disability x iatrogenic mortality of treatment.

One assumption in this is of perfect patient follow-up to minimise relapse but follow-up compliance has been shown to be poor in the DRC [6].

**References**

1. Lutumba P, Robays J, Miaka C, Kande V, Mumba D, et al. (2006) [Validity, cost and feasibility of the mAECT and CTC confirmation tests after diagnosis of African of sleeping sickness]. Trop Med Int Health 11: 470–478. Available: http://www.ncbi.nlm.nih.gov/pubmed/16553930. Accessed 20 February 2014.

2. Fox-Rushby JA, Hanson K (2001) Calculating and presenting disability adjusted life years (DALYs) in cost-effectiveness analysis. Health Policy Plan 16: 326–331.

3. Lutumba P, Makieya E, Shaw A, Meheus F, Boelaert M (2007) Human African trypanosomiasis in a rural community, Democratic Republic of Congo. Emerg Infect Dis 13: 248–254. Available: http://www.pubmedcentral.nih.gov/articlerender.fcgi?artid=2725878&tool=pmcentrez&rendertype=abstract. Accessed 20 February 2014.

4. Fèvre EM, Wissmann B V, Welburn SC, Lutumba P (2008) The burden of human African trypanosomiasis. PLoS Negl Trop Dis 2: e333. Available: http://www.pubmedcentral.nih.gov/articlerender.fcgi?artid=2602597&tool=pmcentrez&rendertype=abstract. Accessed 24 January 2014.

5. Hackett F, Berrang Ford L, Fèvre E, Simarro P (2014) Incorporating Scale Dependence in Disease Burden Estimates: The Case of Human African Trypanosomiasis in Uganda. PLoS Negl Trop Dis 8: e2704. Available: http://www.plosntds.org/article/info:doi/10.1371/journal.pntd.0002704;jsessionid=7DCD2271CE456A1A7B123A2455980B17. Accessed 14 February 2014.

6. Hasker E, Mpanya A, Makabuza J, Mbo F, Lumbala C, et al. (2012) Treatment outcomes for human African Trypanosomiasis in the Democratic Republic of the Congo: analysis of routine program data from the world’s largest sleeping sickness control program. Trop Med Int Health 17: 1127–1132. Available: http://www.ncbi.nlm.nih.gov/pubmed/22809002. Accessed 20 February 2014.
